# Assessing the staffing needs for primary health care centers in Cross River State, Nigeria: a workload indicators of staffing needs study

**DOI:** 10.1186/s12960-021-00648-2

**Published:** 2022-01-28

**Authors:** S. C. Okoroafor, A. Ahmat, M. Osubor, J. Nyoni, J. Bassey, W. Alemu

**Affiliations:** 1 World Health Organization Country Office for Nigeria, Abuja, Nigeria; 2 World Health Organization Regional Office for Africa, Brazaville, Congo; 3 Global Affairs Canada, Abuja, Nigeria; 4 Cross River State Ministry of Health, Calabar, Nigeria

**Keywords:** Health workforce planning, Workload indicators of staffing needs, Health workforce performance, Primary health care workers

## Abstract

**Background:**

A major human resources for health challenge for Nigeria is ensuring the availability and retention of adequate competent health workers in the right mix to provide health care particularly at primary health care facilities in remote and rural communities. This study applied the Workload Indicators of Staffing Need (WISN) method to determine the numbers of nurses, midwives, community health officers (CHOs), community health extension workers (CHEWs), and junior community health extension workers (JCHEWs) required to cope with health care service delivery at primary health care facilities in Cross River State; compare workloads of different cadres at selected health facilities, and identify facilities with highest workload pressure.

**Methods:**

Cross River State in Nigeria has 18 local governments, 196 wards, and an estimated population of over three million people. We used the WISN method to estimate the numbers of nurses/midwives, CHOs/CHEWs, and JCHEWs required to cope with the workload in the 196 ward-level primary health care facilities.

**Findings:**

Basic services provided by nurses/midwives, and CHOs/CHEWs were typical of the primary health care level. They are antenatal care, routine immunization, child welfare clinic, family planning, treatment of minor ailments, assisted and normal deliveries, postnatal care, emergencies, care of tuberculosis patients, and referrals. Findings show that available nurses/midwives for the 196 PHC facilities were 79, and the calculated requirement was 209, WISN ratio of 0.4 and difference of − 130; the existing number of CHOs/CHEWs was 808, the calculated requirement was 1,258, WISN ratio of 0.6, with a difference of − 450; and the number of existing JCHEWs was 258, the calculated requirement was 203, WISN ratio of 1.3 with a difference of 55. Cross River State had only 40% of required nurses and midwives; and 60% of CHOs/ CHEWs needed to provide health services in the ward-level PHC facilities.

**Conclusion:**

The findings from this study indicated marked shortages of needed health workforce particularly nurses and midwives at the primary level of care; and overlap in some of the tasks performed by nurses/midwives, CHO/CHEWs, and JCHEWs.

## Background

Human resources for health (HRH) are indispensable to the functioning of health systems and critical to achieving universal health coverage (UHC), health-related sustainable development goals (SDG), and national health sector goals. Indeed, health systems can only function with health workers; improving health service coverage and realizing the right to the highest attainable standard of health depend on their availability, accessibility, acceptability, and quality [1].

Globally, the health sector requires large numbers of skilled and unskilled health workers to provide safe health services. The numbers of qualified health workers are often inadequate and a major challenge to the global public health community [2]. Hence, effective HRH planning and management are at the fore of the strategic responsibilities of policymakers and health care managers [1].

Threats to health security are greater in African countries, particularly for the poor in rural and remote communities, poverty juxtaposes malnutrition, low access to health services, lack of clean water, and other necessities [3]. Primary health care (PHC) services are important to solving the basic health needs of populations at this level. However, health workers are sometimes reluctant to work at primary health care facilities of rural and remote communities due to inadequate or no housing, electricity, clean water, basic means of transport, and lack of essential equipment to perform functions [4]. These conditions are daunting often leading to exit from the job and the community.

Typically, HRH planning and management strives to have the right numbers of people with the right skills, in the right places, at the right times with the right attitude, doing the right jobs, at the right costs, and with the right outputs [5]. It entails thinking in advance and predicting the numbers and quality of human resources required to provide quality services in the short and medium-term to achieve health system goals [1]. These are done taking into account the health, social and economic needs of health workers.

A major challenge for Nigeria is ensuring the availability and retention of adequate competent HRH in the right mix to provide health care services. Most rural and deprived communities do not have access to basic health care because of insufficient caregivers. Hence, the Nigerian HRH policy focuses on training and equitably distributing frontline health care workers across the federation comprising 36 states, and the Federal Capital Territory, and 774 local government areas (LGAs) [6]. Many of the local governments are rural and remote, and over 50% of the population lives in these areas, the burden of disease is high, and basic social amenities low. Primary health care is the first point of entry into the health system; nurses, midwives, community health officers (CHOs) community health extension workers (CHEWs), junior community extension workers (JCHEWs) provide basic health care services.

The nation faces low stock of health professionals; inequitable distribution of health workers; and disparities in health worker densities between urban and rural communities, geographical location, and among the three levels of health care [6]. This applies to many states in the country including Cross River State.

Reports show that in 2012, Nigeria had 148,291 nurses, and 101,275 midwives; translating to 249,560 nurses and midwives, with a density of 1.5 nurses and midwives per 10,000 population. For the Community Health Practitioners (CHPs), 5986 CHOs, with a density of 0.4 per 10,000 population,; CHEWs, 42,938, density of 2.5 per 10,000 population; JCHEWs, 28,458, density of 1.7 per 10,000 population [7]. Cross River State had the lowest density for nurses and midwives 11.9 to 100,000 population when compared with other States within the region [7, 8].

The Federal Ministry of Health (FMOH) argues that Human Resource for Health Planning (HRHP) is not integrated because of weakness in coordination and lack of integrated planning framework; health facilities use staffing norm based on population ratio; and deployment planning systems are weak in generating and applying results of studies in staff deployment affecting the equitable distribution of available health workers [6, 8].

The Cross River State government and senior health officials at different levels supported the application of the Workload Indicators of Staffing Need (WISN) method to improve HRH planning and management and increase access to health care services for people in rural and deprived communities. This study was to determine the number of frontline health workers, nurses/midwives, CHOs/CHEWs, and JCHEWs required to cope with the delivery of health services at primary health care facilities in Cross River State; compare workloads of different cadres within selected health facilities, and identify facilities with highest workload pressure.

### Workload indicators of staffing need

The World Health Organization introduced the WISN method in the 1990s to enhance processes for planning and managing human resources for health. It is a methodical approach that deepens health care managers’ understanding of services, their delivery, and inherent complexities; and captures perspectives of health workers on the quantity and quality of services. It is a veritable tool that helps health care managers determine the numbers of health care workers required for the workload of a facility, and the workload pressure, using existing health facility data. Useful for staffing decision-making at every level of the health system; simple, acceptable to health service managers; easy for non-medical health managers to understand; and provides realistic practical targets for budgeting and resource allocation.

The implementation process involves eight steps: determining priority health workers and type of health facilities for the study; estimating available working time; defining workload components; setting activity standards; establishing standard workloads; calculating allowance factors; using WISN to determine staff requirements; and analyzing and interpreting WISN results [5]. Today several countries particularly in developing countries are applying WISN to improve human resources for health planning and management [9, 10].

## Methods

### Setting and design

Cross River State is in the Niger Delta region of Nigeria, has 18 LGAs, 196 wards, and an estimated population of over three million people. The study took place between February 2016 and September 2018; and involved a review of health facility records, ministry records, and key informant interviews. Senior health care managers were eager to cover the State, hence we purposively selected 196 ward-level PHC facilities. The study used WISN to estimate the numbers of nurses, midwives, CHOs, CHEWs, and JCHEWs required to cope with the workload at the selected primary health care facilities.

Introducing a new approach to HRH planning and management required sensitization and training of key stakeholders at different levels of the health system. We ensured that health system policymakers and managers at the federal, state, and local government levels understood the WISN method, process of application, and expected benefits. This made the process highly participatory involving senior, middle, and operational health managers. We held sensitization meetings and training for senior officers of the health sector at the federal, state, and local government levels. This ensured that the policymakers and managers understood the rudiments of the WISN process, strengths, weaknesses, and usefulness of results in HRH planning and management.

We constituted and trained three WISN implementation groups: the State Steering Committee (SSC); Technical Task Force (TTF)/WISN Study Group (WSD); and three cadre-specific Expert Working Groups (EWGs)—one group each for the nurses and midwives, CHOs and CHEWs and the JCHEWs. The groups comprised senior administrative officers of the State Ministry of Health (SMOH), State Primary Health Care Development Agency (SPHCDA), PHC Care Coordinators, representatives of health training institutions for nurses, midwives, and community health practitioners; representatives of professional associations; and experienced senior PHC workers still delivering services at the PHC facilities.

Each EWG defined the workload components for a specific cadre—the health services, support services, and additional activities—and set service, category, and individual activity standards for the workload groups. Consensus and pre-validation occurred during plenary sessions. A separate cadre-specific EWG validated the workload components and activity standards.

### Data collection and analysis

The Technical Task Force (TTF) and PHC coordinators conducted a pilot test of the data collection process using (5 randomly selected health facilities in rural and urban communities. The process included a desk review of Cross River State public service rules and key informant interviews using an open-ended questionnaire to obtain information on working hours per day, working days per week, public holidays, approved sick leave days, and the average number of days that health workers are away on training organized by government and development partners. Data on the health service activities were obtained from the national district health information system (DHIS) platform using a questionnaire. This was informed by a previous WISN study that established the DHIS as a reliable and readily available source of health service statistics needed for the conduct of WISN [9]. The pilot process served to validate the identified health service, additional and support activities and activity standards, identify the other data sources for the study and triangulate the statistics from health service activities obtained from the DHIS and national health management information system (NHMIS) monthly summary form (MSF). Following the pilot, no changes were made to the workload activities and standards, and the data collection process and tool were replicated in the 196 ward-level PHCs in Cross River State.

All data inputs were entered in the WISN software with the WISN results obtained.

## Findings

### Available working time, workload components, and activity standards

In Cross River State, nurses, midwives, CHOs, and CHEWs were on duty for 5 days in a week and 8 h per day. They are entitled to 30) days of annual leave and seven (7) days of sick leave. In addition, they also took 13 days to observe public holidays, an average of 2 days of special no notice leave; and 30 days of training. The JCHEWS worked e5 days a week and 8 h per day; are entitled to 21 days annual leave and 7 days of sick leave; and observed 13 days for public holidays, 2 days of special no notice leave; and also, 30 days for training. Based on these, the calculated AWT for nurses, midwives, CHOs, and CHEWS was 35.6 weeks, 178 days, and 1424 h; and that of JCHEWS was 37.4 weeks, 187 days, and 1496 h (Table 1).

**Table 1:** Available working time for nurses, midwives, CHOs, CHEWs and JCHEWs

Cadre	Working days per week	Working hours per day	Annual leave	Public holidays	Sick leave	Special no notice leave	Training days per year	AWT in weeks	AWT in days	AWT in hours
Nurses/midwives	5	8	30	13	7	2	30	35.6	178	1424
CHO/CHEWS	5	8	30	13	7	2	30	35.6	178	1424
JCHEWS	5	8	21	13	7	2	30	37.4	187	1496

*AWT* available working time, *CHO* Community Health Officer, *CHEW* Community Health Extension Worker, *JCHEW* Junior Community Health Extension Worker

Basic services for nurses/midwives, and CHOs/CHEWs were antenatal care, routine immunization, child welfare clinic, family planning, treatment of minor ailments, deliveries (normal and assisted), postnatal care, emergencies, care of tuberculosis patients, and referrals. The time each cadre spent on activities varied, but normal and assisted deliveries by nurse/midwives and CHOs/CHEWs took the longest time from 75 to 160 min per patient. Nurses/midwives, CHO/CHEWs, and JCHEWs were involved and spent nearly the same time on an antenatal clinic, 50, 40, and 30 min per patient; routine immunization, 27, 23, 20 min per patient; and child health clinic, 35, 40, and 42 min per patient. For the treatment of minor ailments, the CHO/CHEWS spent 62 min per patient; and JCHEWS, 61 min per patient spent much longer time than the nurses/midwives, 27 min per patient (Table 2).

**Table 2:** Health service activities and service standards for nurses/midwives, CHOs/CHEWS and JCHEWs

Health services	Service standards for cadres			Unit time
	Nurses/midwives	CHO/CHEW	JCHEW	
Antenatal clinic (ANC)—first visit	50	40	30	Minutes/patient
Antenatal clinic (ANC)—subsequent visits/revisits	21	25	27	Minutes/patient
Routine immunization	27	23	20	Minutes/patient
Child welfare clinic (sick child) U5	35	40	42	Minutes/patient
Family planning—counselled	15	40	43	Minutes/patient
Family planning—oral	5	5	5	Minutes/patient
Family planning—injectable	8	10	12	Minutes/patient
Family planning—insertion (IUCD and implant)	15	16		Minutes/patient
Treatment of minor ailments (children and adults)	27	62	61	Minutes/patient
Delivery (normal delivery)	75	140		Minutes/patient
Delivery (assisted)	90	160		Minutes/patient
Post-natal care (booked case and unbooked)	16	25		Minutes/patient
PMTCT—mothers (booked and unbooked)	14	26		Minutes/patient
PMTCT—infant (booked and unbooked)	23	28		Minutes/patient
Accidents and emergencies—minor cases	20	38	29	Minutes/ patient
Accidents and emergencies—major cases	9	10		Minutes/patient
Care of a patient with tuberculosis (TB)	23	33		Minutes/patient
2-way referrals	35	38	35	Minutes/patient

*CHO* Community Health Officer, *CHEW* Community Health Extension Worker, *JCHEW* Junior Community Health Extension Worker, *PMTCT* prevention of mother to child transmission of HIV

Validated support activities include community mobilization and education, time ranged from 3 h per month for nurses/midwives, 4 h for CHOs/CHEWs, and 8 h per month for JCHEWs. Others were group health education, community development committee, and ward development committee meetings (Table 3). Additional activities for nurses/ midwives, and CHOs/CHEWs were largely performed by heads of units. For instance, nurses/midwives spent 32 h per month on supervision of students, 6 h per month on general administration, and 4 h per month on mentoring subordinates. Other activities were monitoring and evaluation, diverse meetings, reporting, advocacy, and sterilization of equipment (Table 4).

**Table 3:** Support activities and category allowance standards for frontline health workers

Workload components	Actual working time per cadre		
	Nurses/midwives	CHO/CHEW	JCHEW
Community mobilization and education	3 h/ month	4 h/month	8 h/month
Group health education	1 h/ week	1 h/ week	1 h/week
Community development Committees (CDC) meetings	2 h/ month	2 h/ month	2 h/month
Ward development Committees (WDC) meetings	3 h/ month	3 h/month	3 h/month
Outreaches/community-based services	4 h/month	20 h/month	21 h/month
Handing over/taking over, report writing and ward round (inpatient and outpatient)	30 min/day	30 min/day	30 min/day
Follow-up care/ home visits	3 h/week	3 h/week	3 h/week
Staff meetings	2 h/month	2 h/month	2 h/month
Documentation on patients	1 h/month	1 h/month	1 h/month

*CHO* Community Health Officer, *CHEW* Community Health Extension Worker, *JCHEW* Junior Community Health Extension Worker

**Table 4:** Additional activities and individual allowance standards for health workers

Workload components	Nurses/midwives		CHO/CHEW	
	Number of staff performing the task	Actual working time	Number of staff performing the task	Actual working time
Supervision of students	10	32 h/month	10	8 h/month
General administration	1	6 h /month	1	4 h/month
Monthly report writing	1	1 h/month	1	1 h/month
Monitoring and evaluations/documentations/collation of patient data	1	3 h/month	1	3 h/month
Review meetings (LGA coordination meeting)	1	3 h/month	1	3 h/month
Mentoring of subordinates	1	4 h/ month	1	4 h/month
LGA technical meetings	1	2 h/month	1	2 h/month
PHC management committee meeting/facility management meeting	1	2 h/month	1	2 h/month
Advocacy	1	6 h/year	1	10 h/year
Bed making	2	24 min/day	2	24 min/day
Sterilization of equipment’s	1	30 min/day	1	30 min/day

*CHO* Community Health Officer, *CHEW* Community Health Extension Worker

### WISN requirements for nurses/midwives, CHOs/CHEWs, and JCHEWs

Using validated variables on Tables 1, 2, 3, 4, we applied WISN to calculate staffing requirements for nurses/midwives, CHOs/CHEWs, and JCHEWs. Results in Table 5 show that available nurses/midwives for the 196 PHC facilities in the 18 LGAs were 79, and the calculated requirement was 209, WISN ratio of 0.4, and the difference of − 130. The existing number CHOs/CHEWs was 808, calculated requirement 1258, WISN ratio of 0.6, with a difference of − 450; and the number of existing JCHEWs was 258, calculated requirement 203, WISN ratio of 1.3 with a difference of 55. The WISN ratio of the LGAs with a shortage of nurses/midwives varies from 0.0 to 0.7 indicating that they have 0–70% of the required nurses/midwives. For CHOs/ CHEWs, the WISN ratio of the LGAs with a shortage varies from 0.3 to 0.9 indicating that they have 30–90% of the required members of this cadre.

**Table 5:** WISN results for nurse/midwives, CHOs/CHEWs, and JCHEWS

Local government area	Nurse/midwives				CHO/CHEWS				JCHEWS			
	Existing staff	Calculated requirement	WISN ratio	WISN difference	Existing staff	Calculated requirement	WISN ratio	WISN difference	Existing staff	Calculated requirement	WISN ratio	WISN difference
Abi LGA	2	10	0.2	**− **8	33	74	0.4	**− **41	5	6	0.8	**− **1
Akpabuyo LGA	2	10	0.2	**− **8	51	71	0.7	**− **20	7	5	1.4	2
Bekwara LGA	3	11	0.3	**− **8	35	47	0.7	**− **12	11	11	1.0	0
Akamkpa LGA	5	13	0.4	**− **8	28	53	0.5	**− **25	17	17	1.0	0
Bakassi LGA	0	10	0.0	**− **10	16	52	0.3	**− **36	6	8	0.8	**− **2
Boki LGA	6	11	0.5	**− **5	43	62	0.7	**− **19	9	7	1.3	2
Calabar Municipal	5	10	0.5	**− **5	68	78	0.9	**− **10	19	12	1.6	7
Calabar South	0	12	0.0	**− **12	50	90	0.6	**− **40	14	12	1.2	2
Etung LGA	7	12	0.6	**− **5	70	56	1.3	14	21	11	1.9	10
Ikom LGA	6	14	0.0	**− **8	41	92	0.4	**− **51	7	8	0.9	**− **1
Obudu LGA	9	12	0.7	**− **3	34	68	0.5	**− **34	12	13	0.9	-1
Obubra LGA	6	12	0.5	**− **6	34	69	0.5	**− **35	11	11	1.0	0
Obanilikui LGA	4	10	0.4	**− **6	45	53	0.8	**− **8	11	9	1.2	2
Ogoja LGA	2	10	0.2	**− **8	40	63	0.6	**− **23	11	10	1.1	1
Odukpani LGA	0	13	0.0	**− **13	45	94	0.5	**− **49	7	6	1.2	1
Yakurr LGA	4	14	0.3	**− **10	45	119	0.4	**− **74	24	30	0.8	**− **6
Yala LGA	17	14	1.2	3	108	56	1.9	52	54	15	3.6	39
Biase LGA	1	11	0.1	**− **10	22	61	0.4	**− **39	12	12	1.0	0
Total	79	209	0.4	− 130	808	1258	0.6	− 450	258	203	1.3	55

*CHO* Community Health Officer, *CHEW* Community Health Extension Worker, *JCHEW* Junior Community Health Extension Worker

**Fig. 1:**
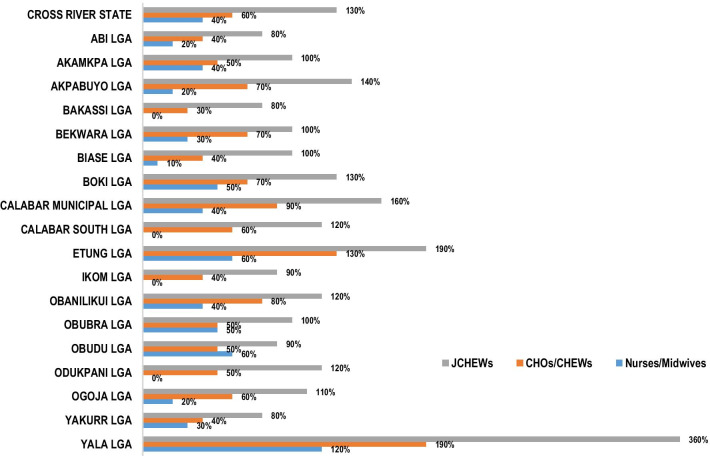
Level of staffing for nurse/midwives, CHOs/CHEWs, and JCHEWS. *CHO* Community Health Officer, *CHEW* Community Health Extension Worker, *JCHEW* Junior Community Health Extension Worker

Some LGAs had excess numbers of JCHEWs, Yala 360%, Etung 190%, Calabar Municipal 160%, Akpabuyo 140%. Yet, Abi, Bakassi, and Yakurr LGAs had only 30% of JCHEWS. Many LGAs had 0–20% of nurses/midwives. Indicating that the workload pressure for this cadre was generally high in Cross River State, yet Yala LGA had 120% of nurses and midwives (Fig. 1).

## Discussion

This study highlights limited numbers of critical frontline health workers at the primary health care level in many local governments that are largely rural and builds on previous findings [9]. Even in countries where the majority of the population lives in rural areas, health care resources are concentrated in the cities [10] and other levels of care [11], this includes health workers. We emphasize the need for nurses and midwives in underserved, remote, and rural communities where maternal and child mortality and morbidity rates are usually high, despite marginal improvements. In this study, 11 (61%) of health facilities had 0–40% of required nurses and midwives. Yet, basic primary health care services are designed to promptly deal with health care needs and save lives. Primary health care is to ensure the highest possible level of health and well-being, by focusing on people’s needs and preferences as early as possible from health promotion to disease prevention to treatment, rehabilitation, and palliative care and close to their everyday environment [12].

Our study identified overlap in some of the workload activities for nurse/midwives, CHO/CHEWs, and JCHEWs. If the three groups were qualified to perform certain tasks and the dire need of a cadre, it is then more effective to transfer such tasks to the cadre in abundance, in line with the task shifting and task sharing of the federal ministry of health. This would ultimately improve access to health care services by clients accessing services at this level. The task shifting and task sharing policy is part of the government’s effort to address workforce shortages in the country and contribute to universal health coverage. This, however, should be implemented with adequate capacity enhancement strategies instituted.

The presence of qualified and motivated human resources is essential for adequate health service provision [13]. Where critical health workers are not available in the community it makes access to health care difficult; affects the quality and quantity of available health services, and impedes progress towards universal coverage. Health workers are inextricably linked to the performance of health systems; and higher job satisfaction leads to higher organizational performance and customer satisfaction. When employees are satisfied with their jobs they are likely to behave toward customers in ways that yield positive service satisfaction [14]. Health workers are also more likely to work where they are needed. Addressing the peculiar needs of skilled health workers particularly those who provide services in remote and rural communities is necessary for retention, high performance, and increase access to the people and quality of service. Nurses and midwives are essential in the delivery of maternal and child health services; and many factors make the nursing profession more vulnerable to stress, intense interpersonal relationship, lack of proper job description, organizational factors, less monetary benefits, overloading, staff shortage, time deficit, and dealing with vulnerable and needy patients [15].

The WISN results provide reliable evidence to guide in the deployment and redistribution of health workers and tasks within health facilities and among the LGAs, based on workloads and local characteristics to promote equity in access and improve the quality of services. The State Primary Health Care Development Agency might consider task shifting and sharing to reduce the workload pressure on nurses and midwives. The participatory completion of the WISN process and emerging evidence, highlighting HRH mal-distribution and inadequacies, provide an opportunity for integrating WISN into the health workforce planning and management policies, strategies, and processes in Cross River State.

Experience shows that often, as access to care increases health systems risk being overburdened and fail to deliver safe, effective, and patient-centered care required for optimal health outcomes at individual and population levels [16]. Positive health outcomes in rural communities require motivated and skilled health workers committed to delivering equitable quality health services. This refers to the absence of disparities in the quality of health services between individuals and groups with different levels of underlying social disadvantage [17], required to improve health indices in African countries.

Furthermore, the health sector subsystems are interconnected and work in synergy to achieve results, understanding the poor health situation in Nigeria requires a holistic approach [18]. At the macro-level, underpinned by the fact that health is deeply interconnected with the environment, trade, economic growth, social development, national security, human rights, and dignity, national health security is a defense against internal and external public health risks and threats that by nature do not respect borders [19]. This implies that health workers are also at the center of national economic growth and security.

## Conclusion

Our findings indicated marked shortages of needed health workforce, particularly nurses and midwives at the primary level of care; and overlap in some of the tasks performed by nurses/ midwives, CHO/CHEWs, and JCHEWs. Since the three groups were qualified to perform certain tasks, it is then more effective to transfer such tasks to the cadre in abundance, in line with the task shifting and task sharing of the federal ministry of health. The task shifting and task sharing policy is part of the government’s effort to address workforce shortages in the country and contribute to universal health coverage. The goal is to achieve universal health coverage and health needs of the population through the mobilization of available human resources to ensure equity, accessibility, and effectiveness in the delivery of essential health care services [20].

The team applied a participatory approach involving major ministry officials, secondary and primary health care officials, and officers from training institutions. We trained adequate numbers of senior health care professionals to continue applying the WISN method and institutionalize the process, for credible HRH planning. The State Ministry of Health may also consider conducting WISN for the secondary health facilities that serve as referral centers to understand the workload pressure.

## Data Availability

Data and materials are available on request.

## Abbreviations

CHEW: Community Health Extension Workers
CHO: Community Health Officers
CHPs: Community Health Practitioners
EWG: Expert Working Group
FMOH: Federal Ministry of Health
HRHM: Human Resource for Health Management
HRHP: Human Resource for Health Planning
JCHEWS: Junior Community Health Extension Workers
LGA: Local Government Area
NPHCDA: National Primary Health Care Development Agency
SMOH: State Ministry of Health
SPHCDA: State Primary Health Care Development Agency
SST: State Steering Committee
TTF: Technical task force
WISN: Workload indicators of staffing needs
WMHCP: Ward minimum health care package
WSG: WISN Study Group
